# Soundscape and fish passive acoustic monitoring around a North Sea gas-production platform in the Dogger Bank

**DOI:** 10.1371/journal.pone.0319536

**Published:** 2025-04-02

**Authors:** Marta Bolgan, Shireen J. Bhalla, Ian Boyer Todd, Victoria L. G. Todd

**Affiliations:** 1 Ocean Science Consulting Limited, East Lothian, Scotland, United Kingdom; 2 Institute of Sound and Vibration Research, Southampton University, Highfield, Southampton, United Kingdom; University of Messina, ITALY

## Abstract

This study provides temporo-spatial characterisation of the underwater soundscape in proximity of a relatively newly installed offshore gas-production platform in the North Sea’s Dogger Bank Special Area of Conservation, recorded by Static Acoustic Monitoring at different distances from the wellhead (70 m, 5 Km and 10 km). Long-Term Spectrogram Analysis and percentile Power Spectral Densities demonstrated strong acoustic similarity between sites; no biophonic acoustic-mass phenomena were present. All locations were characterized by Underwater Radiated Noise, concentrated < 2 kHz, which dominated the soundscape. Fish acoustic community analysis was performed to explore occurrence, richness, abundance, diel, and seasonal patterns of putative fish sounds. Principal Component Analysis was used to infer potential sound-emitting species, and was performed on North Sea fish sounds downloaded from the Global Inventory of known fish sounds (https://fishsounds.net/), analyzed for the same acoustic features used to characterize fish sounds recorded during this study. The fish acoustic community was characterized by low levels of diversity (acoustic richness ranging from 1 to 2) and abundance (never above 2 sounds min^-1^). The fish sound type ‘Pulse Series’ (PS), emitted at the 70 m and at the 5 km station in low abundance in September from ca. 19:00 to 23:00, was characterized by acoustic features with the closest linear combination to those typifying sounds emitted by *Eutrigla gurnardus*. The fish sound type ‘Low-frequency Down-Sweep’ (LF-DS) was recorded at all stations and was characterized by acoustic features with the closest linear combination to those typifying grunts emitted by *Gadus morhua*. This study represents the first application of fish acoustic community analysis in the context of environmental management of an operational offshore gas production platform.

## Introduction

The term ‘soundscape’ was first used at the end of the last century for describing the acoustic environment, and can be defined as ‘*the collection of sounds within an environment at a particular moment in time’* [1,2]. Each localised soundscape results from overlap of three distinct sonic sources: geophony, *i.e.,* noise produced by non-biological natural sources, biophony, *i.e.,* sounds emitted by living organisms, and anthrophony, *i.e.,* noise associated with human activities [1]. Soundscape analysis considers all simultaneous sonic sources within the environment where data are collected (depending on sample rate), and provides non-invasive, cost-effective, scalable, high-resolution temporo-spatial information about animal populations, ecosystem processes, status, dynamics, and human impacts [3–6].

Soundscape recordings can be used to document the global state of sound-producing life and the environment, thus informing conservation actions, biodiversity stewardship, protected area management, and potentially soundscape-based policies [7]. As such, soundscape analysis can support the 15^th^ Conference of the Parties (COP15) biodiversity monitoring targets within the Global Biodiversity Framework and track progress towards United Nations (UN) Sustainable Development Goals [7]. While current academic research focuses on soundscapes and their biological components [e.g., 3, 4, 6–10], at the legislative level, soundscapes are just beginning to be documented as important ecosystem features to be considered, such as airborne soundscapes recognised by the Welsh Government Noise and Soundscape Action Plan, https://www.gov.wales/noise-and-soundscape-action-plan-2018-2023-0 [11].

Aquatic animals rely on information encoded in soundscapes for activities which are crucial for their survival such as orientation [12], navigation [13], mate searching [14], spawning activity [15], foraging [16], territorial defense [17], and predator avoidance [18]. Consequently, these biological processes, in turn, actively shape soundscapes. In fish, acoustic communication has evolved independently more than 30 times, occurs potentially in nearly two-thirds of ray-finned species, and is associated with vital biological processes, such as spawning, courtship, mate choice, feeding, and competition for limited resources [19–22]. Consequently, in aquatic ecosystems, ranging from inland waters to tropical coral reefs, from temperate coastal areas to underwater seamounts, canyons and abyssal planes, fish-communicative sounds constitute an integral part of underwater soundscapes [5,20,23–28].

The shallow (<50 m) Dutch North Sea’s relatively homogenous sandy seabed is one of the busiest maritime areas in the world and, accordingly, is a noisy place [29–31]. Aside from hosting the largest European port (Rotterdam), other human activities compounding the underwater soundscape include, *inter alia,* heavy commercial fisheries, busy ferry lines between England and the Netherlands, construction and operation of wind farms, detonation of unexploded World War II ordnance, and Oil and Gas Exploration and Production, O&G E&P, *etc.* [29,30,32–34]. Sertlek et al. [29] mapped the temporo-spatial-spectral distribution of noise radiated by several anthropogenic sources (*e.g.*, ships, seismic airguns, explosives, *etc.*) in the Dutch North Sea. This study showed that anthrophony is responsible for one-hundred times more acoustic energy (averaged over two years) than naturally-occurring geophonic sources, such as wind [29]. Basan et al. [30] demonstrated that, across the whole North Sea, Underwater Radiated Noise (URN) emanating from vessels is responsible for the highest Sound Pressure Levels (SPLs) across the 10 Hz – 20 kHz bandwidth; in particular, URN is concentrated in the 50 Hz – 1,250 Hz band and peaks between 100 Hz and 500 Hz [35–37]. Despite these—and other studies documenting specific anthropogenic sources occurring in the Dutch North Sea [29,32,33,38]—to the best of our knowledge, no study to date has investigated the presence of fish communicative sounds in the context of environmental management of an operational offshore gas production platform.

Owned and operated by Petrogas E&P, the relatively new, predominantly unmanned A18 satellite-offshore-gas-production platform was installed on 4^th^ October 2015 in the Dutch sector of the North Sea, *ca.* 300 km north of Den Helder, Netherlands, in a water depth of *ca.* 47 m. Perched on the border of the Dogger Bank Special Area of Conservation (DBSAC), the regulatory authorities supported environmental monitoring studies using incidentally collected Remotely Operated Vehicle (ROV) data and Passive Acoustic Monitoring (PAM) using C/F-PODs to assess the platform’s effect on marine life (predominantly invertebrates, fish, and marine mammals) during its initial construction [39], and subsequent operation [40–42]. Studies showed that during A18 construction, there was rapid initial ‘colonisation’ by fish communities such as common dab *Limanda limanda* (L., 1758), common dragonet *Callionymus lyra* (L., 1758), whiting *Merlangius merlangus* (L., 1758), and grey gurnard *Eutrigla gurnardus* (L., 1758), with increases in species richness (S), abundance (N), and diversity (H’) over the four-day (entire) study period [39]. In terms of negative effects, during the platform’s construction, acoustic presence of harbour porpoise (*Phocoena phocoena*) reduced significantly compared to baseline (pre-platform-presence); however, activity returned to baseline within three months [41]. Naturally, on a new ‘clean’ platform, these species were considered to be ‘visitors’ to the new ‘artificial reef’; however, it is worth noting that while whiting are unlikely to emit sounds due to the absence of sonic muscles [43], grey gurnard has been documented as soniferous [19]. The second wave of ‘colonisation’ study [40] presented greater diversity than the pioneering community, and included the same species noted in the first study, and other commercial species, including Atlantic cod *Gadus morhua* (L., 1758), which is a well-known vocal species [43]. Further commercial species identified were bib (*Trisopterus luscus* L., 1758), horse mackerel (*Trachurus trachurus* L., 1758), and a new North Sea record of a pompano (*Trachinotus ovatus* L., 1758). Carangidae species (such *T. trachurus* and *T. ovatus)* have been reported as acoustically active; however, no study to date has validated the possibility of recording their communicative sounds in natural environments [44,45].

The aim of this study was to provide temporo-spatial comparison of the underwater soundscape characterising the proximate and far-field vicinity of the relatively new A18 gas-production platform, with a particular focus on presence of fish sounds. It is hypothesised that, because of the relative newness of the platform, temporo-spatial patterns in acoustic levels, energy distribution across frequencies, and soundscape components are likely to be similar at the A18 platform, compared to control locations. Furthermore, it is hypothesised that fish-acoustic richness and abundance are likely to be similarly low at all locations, due to high anthropogenic pressures characterising the Dutch North Sea in general.

## Materials and methods

### Timing & location

The study took place from 21^st^ July until 13^th^ November 2022. Full site description and A18 platform details can be found in Todd et al. [39]. Experimental design followed that of Todd et al. [41], as this soundscape study formed part of a wider, long-term (since 2015) ongoing underwater noise and marine mammal study at the A18 platform; consequently, mooring locations were fixed for experimental standardisation. Underwater soundscape was thus monitored simultaneously in three recording sites located at different distances from the A18 gas-production platform: 70 m away from the A18 wellhead, and 5 km and 10 km away, in a northeasterly direction (see **Table 1** for deployment details).

**Table 1:** A18 platform recording coordinates in World Geodetic System (WGS) ’84 degrees and decimal minutes. Date in day, month, year.

Site name	Lat (N)	Long (W)	Distance from A18 (m)	Deployment date	Retrieval date
70 m NE	55˚06.317’ N	003˚49.974’ E	70	21/07/2022	12/11/2022
5 Km NE	55˚08.174’ N	003˚53.330’ E	5000	21/07/2022	12/11/2022
10 Km NE	55˚10.062’ N	003˚56.670’ E	10000	21/07/2022	13/11/2022

Experimental justification of mooring positions is described elsewhere in Todd et al. [41], however, in brief: control locations were selected 5 km and 10 km away from the platform based on the following criteria: 1) sufficient distance from the A18 platform to minimise recording the same soundscapes at all locations simultaneously (*i.e.,* avoid autocorrelated data sets); 2) not be influenced by any potential low-frequency jack-up drilling-rig-operational sounds (should a jack-up rig attach to the platform during the study duration, which at the time of the study was not known, but later confirmed to not be the case), based on noise-spreading-loss calculations derived from 2004 noise-level and frequency measurements of the *Noble Kolskaya* exploration jack-up drilling rig at block B4-05, 67.4 km NE of A18, also on the DBSAC [38]; 3) Minimal differences with respect to topography, water depth, bottom type, fauna, and flora, and oceanographical conditions, which was established by consulting surveys [46,47], and Conductivity, Temperature, Depth (CTD) profiling (V. L. G. Todd *in. prep.*); and, 4) distances further than 5 and 10 km risked placement of moorings in different weather cells or ecological habitats, or closer to other operational O&G fields and their associated soundscapes.

### Equipment & data collection

At each site, the underwater soundscape was recorded using an underwater noise recorder (Porpoise, Turbulent Research Inc., Bedford, CA), with a hydrophone sensitivity of -193 dB re 1 V/μPa, a flat frequency response of 2 Hz to 384 kHz, and a sample rate of 48,000 Hz, 24 bits. wav. Prior to deployment, noise recorders were synchronised in the laboratory and programmed to start a few days after deployment (to avoid recording positioning noise); instruments were set to record continuously (29 min wav. files). Recorders were moored to the seabed using acoustic releases (Sonardyne, Aberdeen, UK). Instruments were deployed at all sites continuously for a period of five months from 20^th^ July 2022 to 12^th^ November 2022 (see **Table 1** for details).

### Acoustic analysis

Soundscape variability between sites was analysed using a twofold approach. The first part (called ‘soundscape analysis’ below) employed Long-Term Spectrograms Analysis (LTSA,) and percentile Power Spectral Density (PSD), calculated on the whole sample rate (*i.e.,* 48 kHz, acoustic data up to 24 kHz) to investigate acoustic-mass phenomena (*e.g.,* animal choruses, *sensu* Cato [48]) and to evaluate diel, seasonal, and spatial (*i.e.,* between locations) patterns of acoustic sources (both biological and anthropogenic). The second part (termed ‘fish-sound analysis’) sought to investigate occurrence, and potentially identify (at specific level), transient signals which could be attributed to fish-communicative sounds.

### Soundscape analysis

Long-Term Spectrograms Analysis (LTSA) provide spectral information averaged over time, which are particularly useful when persistent spectral features are under investigation [49]. Consequently, they are effective visualisation tools for evaluating spectral and temporal variations in long-duration audio recordings [4]. Their efficacy in delineating soundscape heterogeneity has been discussed and proven by different studies [4,5]. In this paper, broadband LTSA of all acoustic data collected in the three locations (**Table 1**) were created and inspected at monthly scale using MATLAB R2023R (Triton package https://www.cetus.ucsd.edu/technologies_triton.html, sample rate 48 kHz, Hanning window, FFT 4,800; frequency resolution 10 Hz, temporal resolution 5 s).

Following this initial macroscopic-acoustic screening based on monthly LTSA, six days per month per location (1^st^, 5^th^, 10^th^, 15^th^, 20^th^, 25^th^ of each month, with the exeption of July and November 2022, depending on deployment and retrivial days, total analysis time =  1656 h of recordings) were selected for further analysis; this temporal subsampling covered the full month and afforded the capture of diversity and variability of fish-acoustic commuities [50]. All subsequent analyses were based on these same six days of recordings processed for each month and site. LTSA and PSD percentile analysis on this data subsample were created in MATLAB R2023R (PAMGuide package https://sourceforge.net/projects/pamguide/ Merchant [51]. Data were downsampled at a sample rate of 8 kHz; LTSA and PSD analysis was carried out using a Hanning window, Fast-Fourier-Transform (FFT) 1,024 (frequency resolution 8 Hz, 50% overlap, Welch factor 10). In particular, PSD data were calculated for the 0- 4 kHz band with a frequency resolution of 8 Hz; these data were imported in R where avereged percentiles were calculated per month and were displayed as a monthly multi-location panel (Rstudio 4.2.1, package tidyverse).

### Fish-sound analysis

Manual analysis was carried out on the above mentioned six days *per* month and *per* site by audio-visual assessment using Raven Pro 64 1.5 (Bioacoustic Research Program, Cornell Laboratory of Ornithology, Ithaca, NY, USA, sample rate 48 kHz, FFT size 3,200 points, 50% overlap, Hanning window) [25]. All sound types with features similar to those characterising sounds emitted by known vocal fish species were considered; these characteristics include, between others, low- frequency content (below 1 kHz), quick pulse repetition rate between pulses of the same sounds and a pulse envelop resembling that of described fish sounds. All fish sound types were categorised using a dichotomous framework – proposed originally by Desiderà et al. (2019) for coastal rocky reefs, and further adapted for different environments [50,52–56].

Hourly data were analysed manually for acoustic richness (*i.e.*, number of sound types) and sound abundance (*i.e.,* abundance of each sound type per minute); for every sound type, a subsample of sounds with a good Signal to Noise Ratio, SNR (SNR > 10 dB, *n* =  28 Low-Frequency Down-Sweep, LF-DS, and *n = * 18 Pulse Series, PS) was analysed for fine intrasound spectral and temporal features (**Table 2**).

**Table 2:** Measured acoustic features on fish sound types.

Sound-feature category	Acoustic feature	Unit of measurement	Acoustic-feature definition
**Temporal**	Sound duration	s	Time interval from peak of first pulse to peak of last pulse
	Number of pulses	--	Number of sharp increases of acoustic intensity or number of cycles of amplitude modulation, *i.e.*, number of basic units composing fish sounds
	Pulse period	s	Time interval between peaks of two consecutive pulses in a sound
**Spectral**	Peak frequency	Hz	Frequency with the highest energy
	Frequency 5%	Hz	Frequency that divides spectral content into two intervals containing 5% and 95% of the energy
	Frequency 95%	Hz	Frequency that divides spectral content into two intervals containing 95% and 5% of the energy

To attempt specific identification of any recorded fish sound types, a list of North Sea fish species was downloaded from Fishbase (https://www.fishbase.se/identification/RegionSpeciesList.php?resultPage=2&e_code=139) on 11^th^ May 2023 (see **S1 Table**); this was compiled with information about sound-production mechanisms and characteristics (based on a systematised-literature revision technique, see Rice et al. [22], Fine and Parmentier [57], Parmentier and Fine [58], Looby [59]) and was further integrated with information in Todd et al. [39], Todd et al. [40]), *i.e.,* which fish species have been sighted previously at A18 (see **S1 Table**). A total of 160 bony fish species can be found in the North Sea (fishbase.se, see **S1 Table**); of these, 22 species have been documented to rely on acoustic communication. Another five species are considered most likely to be vocal because of either anedoctical evidence of sound production or morphological investigations which highlighted presence of sound production mechanisms (see **S1 Table**).

All sound files available in the Global Inventory of Fish sounds https://fishsounds.net/ [60] corresponding to soniferous North Sea fish species sighted at the A18 platform [39,40] were downloaded in May 2023 (.mp3), and were analysed for the same temporal and spectral intra-sound features reported in **Table 2**. These sound features were used as variables for Principal Component Analysis (PCA), performed in Rstudio 4.2.1 (package FactoMineR). PCA was run on the correlation matrix to infer potential sound emitting species on both sound extracted from field recordings conducted at the A18 platform, and all known North Sea fish sounds downloaded from fishsound.net (N_sound_ =  165, see **S1 Table**). Scree and score plots for the first two components (which cumulatively explained 67% of variance) were generated (see **Figs S1** and **S2****).**

## Results

### Soundscape

**Fig 1:**
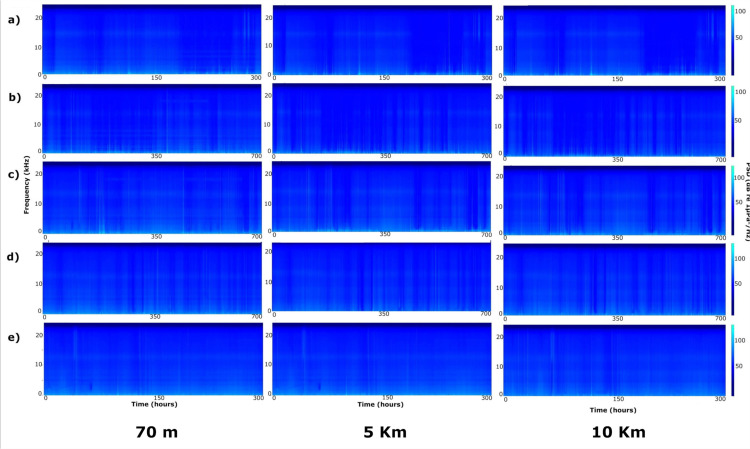
Monthly Long-Term Spectrogram Average visualizing soundscape in the 0 – 24 kHz band recorded at 70 m station, 5 Km station and 10 Km station from July to November 2022; a) July; b) August; c) September; d) October; e) November. July and November are shorter due to deployment and retrieval times.

**Fig 2:**
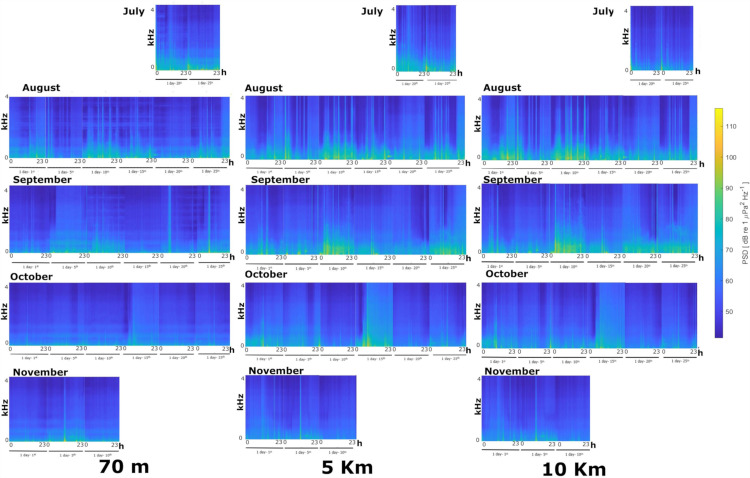
Daily Long-Term Spectrogram Average (six days per site and per month, July and November dependent on deployment and retrieval times) visualizing the soundscape in the 0 – 4 kHz band (y scale upper limit) at the 70 m station, 5 Km station and 10 Km station, from July to November 2022.

**Fig 3:**
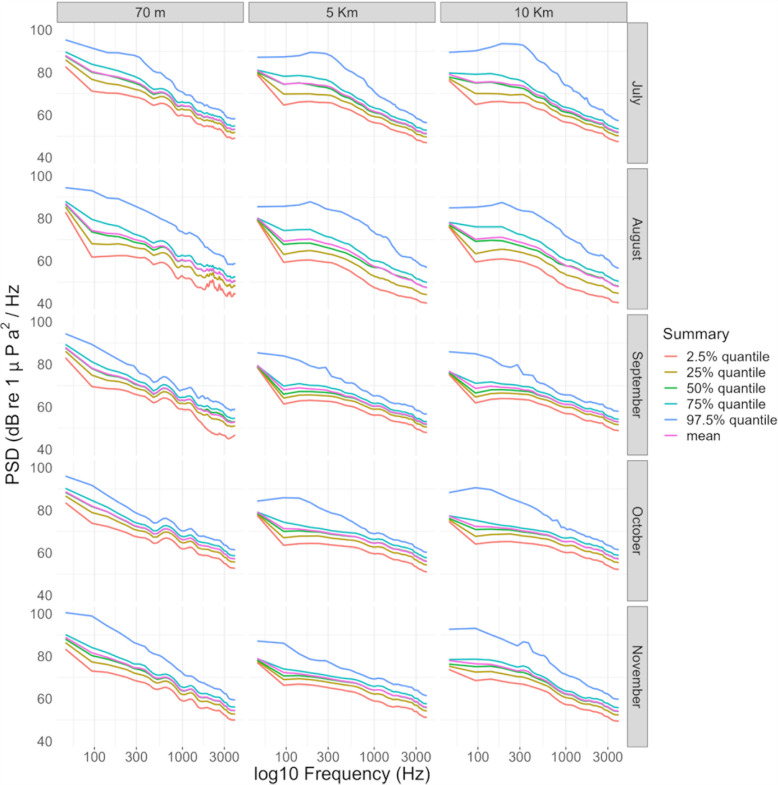
Power Spectral Density plots with cumulative probability distributions of the 2.5%, 25%, 50%, 75% and 97.5% per month and per station (0 – 4 kHz band, log scale for the x-axis).

Long Term Spectrogam Averages and percentile PSD (Figs 1–3) showed strong acoustic similarity between sites, as well as absence of diel and macro seasonal patterns of acoustic sources.

In particular, no acoustic-mass phenomena (*e.g.,* fish choruses and/or low frequency cetacean vocalisations, invertebrate snapping, etc.) was present (Figs 1–3). All locations were characterised by presence of URN [27] concentrated mainly below 2 kHz, peaking between 300 and 500 Hz, which dominated the soundscape at all stations (**Fig 3**). Anthropogenic noise did not show a specific temporal pattern (Fig 1–Fig 3), but was present especially during summer months, *i.e.,* July to September (**Fig 3**).

### Fish-acoustic community

The fish-acoustic community, at all stations, was characterised by extremely low diversity and abundance. Besides the absence of acoustic-mass phenomena (*i.e.,* choruses, *sensu* Cato [48] highlighted by LTSA analysis (Figs 1–3), low acoustic richness and acoustic abundances were detected at all sites and times by manual detection, classification and counting of fish sounds.

**Fig 4:**
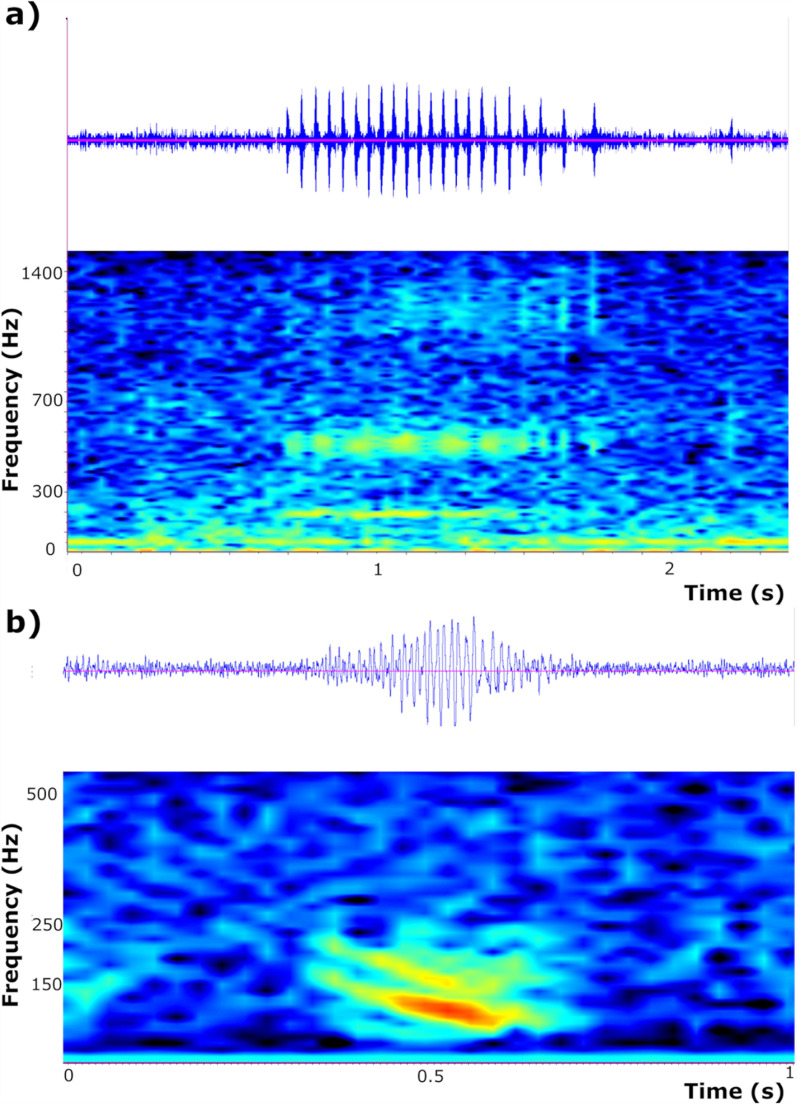
Waveforms and spectrograms of fish sound types recorded during this study a) Pulse Series (PS) and b) Low-Frequency Down-Sweep (LF-DS) (Hanning window, FFT size 3,200, frequency resolution 15 Hz, 50% overlap).

**Fig 5:**
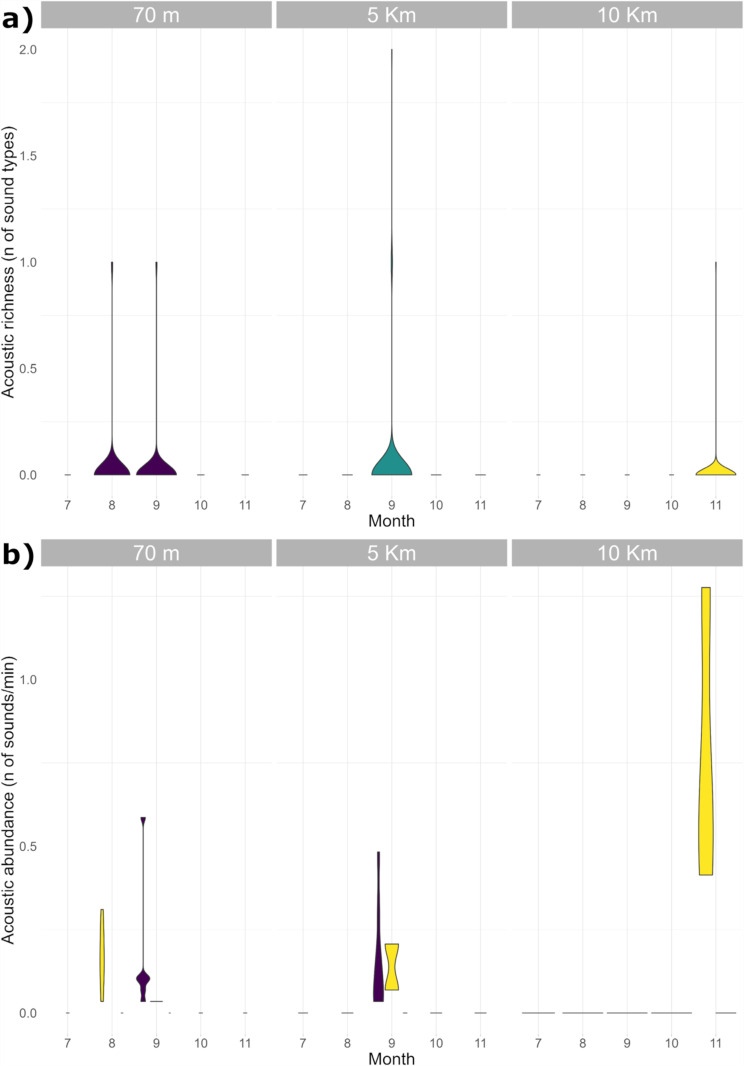
Acoustic richness (number of fish sound types; panel a) and acoustic abundance (number of sounds per sound type, panel b) per month and per station. In panel a) color code identifies the station (purple =  70 m; green = 5 Km; yellow =  10 Km) while in panel b) color code identifies sound type (yellow =  LF-DS Low-Frequency Down-Sweep; purple =  PS Pulse Series). The number on the x-axis represent months (January = 1, February = 2 etc.). The wideness of the violin plots display data density (Kernel density estimate). The extreme values represent data range.

Only two fish sound types were recorded during this study (**Fig 4**); acoustic richness (i.e., number of fish sound types) ranged from 1 (10 Km station) to 2 (70 m and 5 Km station); in the hours in which fish sounds when emitted, sound abundances were never above 2 sounds min^-1^ (maximum abundance: 1.3 ±  0.23 sound min^-1^) (**Fig 5**).

**Fig 6:**
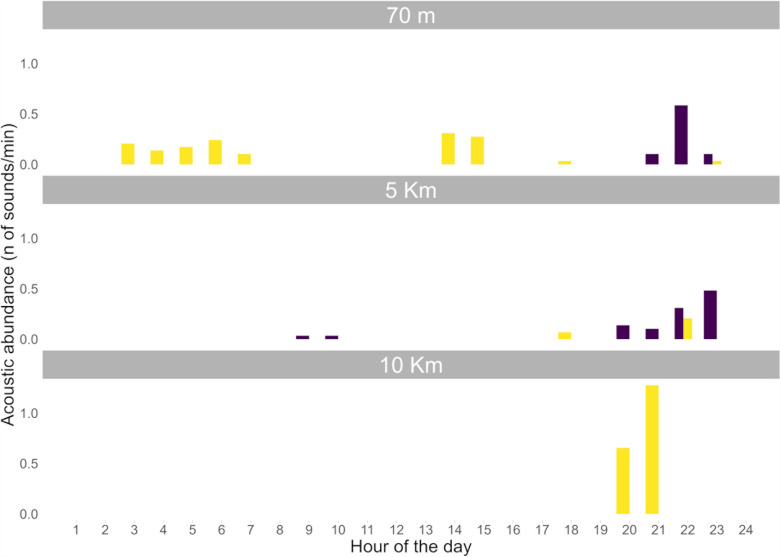
Diel pattern of occurrence of fish-sound type, per hour and per station. Color code identifies sound type (yellow =  LF-DS Low-Frequency Down-Sweep; purple =  PS Pulse Series).

The fish sound type recorded at 70 m and 5 Km station was named Pulse Series (PS; see, e.g., Desiderà et al. [25]; Di Iorio [53]; Bolgan et al. [50], Puebla-Aparicio et al. [54] for definitions and classifications). PS (**Fig 4** and **Table 3**) was detected in September 2022 primarily from 19:00 to 23:00 Universal Time Coordinated (UTC), in low abundance (average of ca. 0.2 sound min^-1^ in the hours in which it was emitted) (**Fig 6**).

**Table 3:** Descriptive statistics of features characterizing fish sound types, i.e., LF-DS =  Low-Frequency Down-Sweep, and PS =  Pulse Series. SD =  Standard Deviation; CV = Coefficient of Variation.

	Freq. 5% (Hz)	Freq. 95% (Hz)	Peak Freq. (Hz)	Duration (s)	Number of pulses	Pulse period (s)
**PS (*n* = 18)**						
Min	128.9	1242.2	527.3	0.543	10.0	0.031
Max	386.7	1910.2	562.5	1.147	37.0	0.063
Mean	330.7	1754.6	543.0	0.874	20.6	0.049
SD	54.91	170.44	9.57	0.21	7.52	0.01
CV (%)	16.6	9.7	1.8	26.6	36.4	13.7
**LF DS (*n = * 28)**						
Min	11.7	164.1	70.3	0.085	10.0	0.005
Max	93.8	914.1	187.5	0.365	52.0	0.013
Mean	65.3	559.6	106.7	0.232	26.3	0.009
SD	15.51	157.23	25.92	0.17	9.32	0.01
CV (%)	23.7	28.1	24.3	29.5	35.3	25.2

The fish-sound type recorded at all stations was named ‘Low-frequency Down-Sweep’ (LF-DS; see, e.g., Desiderà et al. [25]; for definitions and classifications) (**Fig 4** and **Table 3**). LF-DS was recorded in August and September 2022 at the 70 m station; here, it was emitted throughout the day without a specific diel pattern (**Fig 6**). On the other hand, LF-DS was recorded in September at the 5 Km station and in November 2022 at the 10 Km station. At both sites, LF-DS was emitted from ca. 18:00 to 22:00 UTC (**Fig 6**).

**Fig 7:**
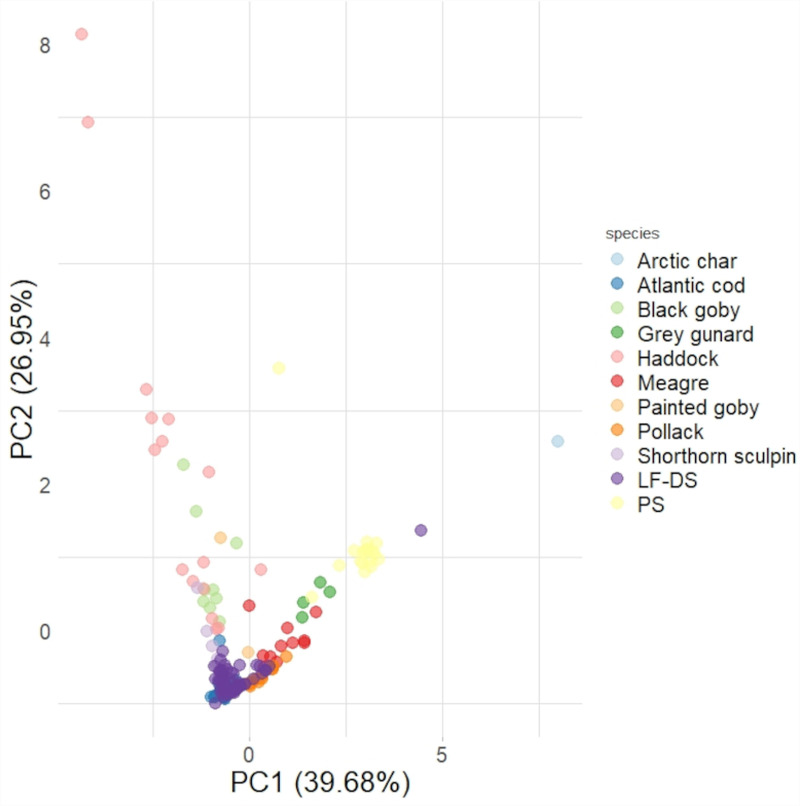
Score plot of Principal Component Analysis (PC1 and PC2) carried out on all fish sounds analyzed as part of this study (Nsound = 165). Variables: 5% Frequency, peak frequency, 95% frequency, number of pulses, duration, and pulse period. Color coding indicates sound-type categories. LF-DS = Low-Frequency Down-Sweep and PS = Pulse Series. North Sea fish species which sounds were downloaded from fishsound.net include; Arctic charr (Salvelinus alpinus L, 1758), Atlantic cod (Gadus morhua L., 1758), Black goby (Gobius niger L, 1758), Grey gurnard (Eutrigla gurnardus Fraser-Brunner, 1938), Haddock (Melanogrammus aeglefinus L, 1758), meagre (Argyrosomous regius Asso, 1801), Painted goby (Pomatoschistus pictus Malm, 1865), Pollack (Pollachius pollachius L. 1758) and Shorthorn sculpin (Myoxocephalus scorpius L. 1758).

Finally, PCA was used to attempt species-specific identification of the fish sound types LF-DS and PS recorded during this study. PCA was performed on LF-DS and PS recorded with a sufficient SNR and on North Sea fish sounds downloaded from the Global Inventory of fish sounds (fishsound.net) *(n* = 165). PCA showed that 67% of variance is explained by spectral features such as peak frequency and frequency quartiles (PC1) and temporal features such as duration and pulse period (PC2) (Fig 7; **Table 4**, see **S1 Fig** and **S2 Fig**).

**Table 4:** Principal Component Analysis (PCA) carried out on all fish sounds analysed as part of this study (*N*_*sound*_ =  138); relevant coefficients, eigenvalues, percentage of the variance and cumulative percentage of the variance explained by the first four PCs.

Variable	PC1	PC2	PC3	PC4
Freq. 5% (Hz)	0.93	0.22	0.13	0.06
Freq. 95% (Hz)	0.89	0.24	-0.01	-0.01
Peak Freq. (Hz)	0.91	0.18	0.12	-0.05
Duration (s)	-0.34	0.86	0.07	-0.37
Number of pulses	-0.40	0.69	0.52	0.31
Pulse period (s)	-0.03	0.58	-0.80	0.18
Eigenvalues	2.8	1.7	0.93	0.28
Percentage (%)	46	28	15	5
Cumulative	46	74	90	95

The PS was characterised by acoustic features with the closest linear combination to that typifying sounds emitted by the grey gurnard (**Fig 7**). The LF-DS was characterised by acoustic features with the closest linear combination to that characterising grunts emitted by Gadidae species such as the Atlantic cod and pollack (**Fig 7**).

## Discussion

This study characterised temporo-spatial underwater soundscape around the relatively newly installed A18 offshore gas-production platform located in the Dutch sector of the North Sea’s Dogger Bank Special Area of Conservation (DB SAC), with particular focus on the fish biophonic component. The underwater soundscape was monitored simultaneously using autonomous acoustic recorders positioned at 70 m away from the A18 platform wellhead, and at further distances of 5 km, and 10 km. All sites exhibited high acoustic similarity in terms of levels, energy distribution across frequencies, and soundscape components. At all stations, the fish-acoustic community was characterised by an absence of acoustic-mass phenomena, and by extremely low levels of acoustic richness and abundance. All locations were typified by presence of URN concentrated < 2 kHz and peaking between 300 and 500 Hz, which dominated the soundscape at all stations and in all seasons. While the clear absence of macro-diel and seasonal patterns in soundscape levels and fish biophonical component found in this study is generally atypical for other marine environments, results are in accordance with what was hypothesised for the experimental design, platform’s age, and North Sea study location.

The North Sea is one of the most industrially active marine environments on Earth [61] and this is clearly reflected by both the overall soundscape and the acoustic fish community. In particular, results presented in this study are in accordance with the study of Basan et al. [30], both in terms of temporal patterns, acoustic levels, and of energy distribution across frequencies. The North Sea soundscape is in fact characterised by negligible seasonal and diel fluctuations; radiated noise emanating from vessels can be considered the root cause of temporo-spatial uniformity of acoustic levels reported in this study. In the Dogger Bank, cargo and fishing vessels are responsible for URN, followed by tankers and other types of vessels [30]. Chronic, long-term effects related to continuous noise such as URN may alter animals’ physiology, behaviour and, ultimately, fitness [62–64]. Furthermore, it is important to underline that environmental stressors such as URN rarely act in isolation. Recent studies demonstrate complex, synergic, cumulative effects of man-made noise and chemical pollution; this warrants further considerations of how combination of sound exposure and other pollutants modifies risks posed as compared to each driver occurring in isolation, at all taxonomic levels [65]. This consideration is particularly important in environments exposed to multiple, concomitant stressors such as the North Sea, and in the context of Environmental Impact Assessments of marine activities.

In this study, the fish acoustic community was characterised by lack of acoustic-mass phenomena at all sites. All locations were characterised by extremely low levels of diversity and abundance; fish acoustic richness (*i.e.,* number of fish sound types) ranged from 1 (10 Km station) to 2 (70 m and 5 Km station); in the hours in which they were emitted, fish sounds abundance were never above 2 sounds min^-1^ (maximum abundance: 1.3 ±  0.23 sound min^-1^). Previous studies on biological species presence at the A18 platform were conducted using ROV images and/or C-POD [39–41]; while images provided evidence of some fish species present at the platform, C-POD demonstrates the presence of harbour porpoises clicks (*Phocoena phocoena*). The evidence that some fish species are present at the platform is not a guarantee of a stable fish community (*i.e.,* spawning grounds). As for harbour porpoises clicks, their main energy is concentrated in frequencies well above the sampling rate of the available recordings (i.e., sample rate of 48 kHz, harbour porpoises click ca. 150 kHz). This explains why harbour porpoises were not detected in this study.

The biophonic component of underwater soundscape is linked to community diversity and stability [4] and to environmental features [53,55]. For example, Staaterman [66] compared acoustic data to visual surveys and found a relationship between ‘low band’ frequencies (<1000 Hz) and the richness and abundance of fish. Desiderà et al. [25] found a relationship between richness and diversity of fish sound types and taxonomic diversity in temperate rocky reefs. In regions not comparable to those of the A18 platform, such as temperate deep-sea environments, the fish acoustic community lacks acoustic-mass phenomena but acoustic richness can be up to ten [52]. In environments similar to those characterising the A18 gas production platform and its surrounding areas, *e.g.,* temperate sandy/muddy habitats, fish acoustic richness is generally much lower (*i.e.,* three) but acoustic mass phenomena are present [67]. Acoustic-mass phenomena are also observed in abyssal planes [5], and in temperate seagrass meadows [50], where acoustic richness can reach up to eight. If we assume that the A18 platform acts as a hard structure, it is worth comparing the findings of this study with those reported for temperate rocky reefs; here, acoustic richness is generally higher than in temperate sandy or muddy areas (i.e., richness can reach up to 13) and fish acoustic-mass phenomena are present [25]. In temperate coralligenous reefs, which are known as biodiversity hotspots, fish acoustic richness can spike up to > 20, while in tropical coral reefs values > 40 are not uncommon [53–55]. While it would be worth comparing results in this study to those of other offshore platforms, and comparable man-made structures (*e.g.,* harbours, bridges, windfarms, *etc*.), there is a marked absence of such studies in the literature. This is because operational-specific noises during all phases are not factored into design of appropriate EIAs, and when formulating associated guidelines and requirements, governmental regulators are faced with limited burden-of-proof baseline data sets, in order to make informed decisions about species statuses, trends and sensitive habitats, including those that are rare or particularly vulnerable to noise impacts. Consequently, EIAs do not currently consider effects of offshore platform presence on species in any context other than short-term noise mitigation context (*e.g.,* noise measurements made during piling of the platform legs); therefore, Before-After-Control-Impact (BACI) studies are lacking, especially with regards to fish acoustics. Nonetheless, it is still possible to deduce that the lack of acoustic-mass phenomena and extremely low levels of acoustic richness and abundances highlighted in this study are due to the heavy, historical, multifaceted human pressures acting in the North Sea, from fishing, to shipping, to energy production [30], and the fact that the platform is still relatively clean, in terms of its faunal colonization, especially breeding fish species.

Despite being highly exploited, the North Sea is productive in term of commercial fisheries, such as Atlantic cod, with hot spots of egg production (spawning grounds) in the southern and eastern edges of the Dogger Bank [68]. Atlantic cod are highly vocal [43], and their acoustic communication and spawning grounds have been monitored in other parts of the world for decades using PAM [69]. Both sexes produce a low-frequency grunt during agonistic interactions, composed of rapidly repeated pulses; grunts—whether single or repeated in a short series—can be emitted during the whole year, but they are produced mostly by males during spawning [43]. Male Atlantic cod hold territories, maintain a size-based-dominance hierarchy, and during spawning, loudness of male sounds is related positively to fish size and dominance [43]. Acoustic-visual-multimodal communication allows sound producers to dominate and exclude less vigorous males and immature/non-breeding females from their vicinity, ensuring a clear area around each dominant male, thus maximizing fitness and reproductive success [43]. Considering Atlantic cod reliance on acoustic signals, its reported presence in southern and eastern edges of the Dogger Bank (Fox et al., 2008), and the results of the PCA here presented, it is likely that LF-DS recorded at the 10 Km station was emitted by Atlantic cod. The low abundance of sounds suggest that the breeding season was not in full swing at any site, which is consistent with the time of recordings (*i.e.,* August to November, *versus* cod spawning season in the North Sea: December to February, Fox et al. [68]). Regarding the lack of a LF-DS diel pattern of detection at the 70 m site, it is possible to hypothesize that presence of sounds during day-time hours may have been influenced by the 24 hr/365-day lighting of the platform itself.

In contrast, less information is available for sound production of the grey gurnard. This species has been reported to emit three types of sound during competitive feeding, which were named knocks, grunts, and growls [70]. The PS recorded in this study is comparable to the growl reported by Amorim et al. [70], both in terms of spectral and temporal features. It is therefore possible to suggest that grey gurnard feed in proximity to the A18 platform and at the 5 Km station.

Remote sensing technologies such as PAM are skewed by the possibility of recording only those species with active sound production; however, it is important to underline that previous studies have suggested that monitoring certain vocally active fish species can provide indirect information over the whole fish community associated with them [71,72]. Passive Acoustic Monitoring is therefore increasingly applied to global study of the ecological impacts of human activities on both ecosystem and biodiversity [3,4,6,7,73]. Long-term studies are necessary for providing an exhaustive, high-resolution, and dynamic picture of maritime activities in the context of environmental management. Acoustic based environmental monitoring inevitably produces large amounts of data, that need to be analysed quickly; manual analysis as that proposed in this study would not be feasible in such contexts. Machine Learning (ML) applications are increasingly applied for solving the intrinsic technical challenge of quickly analysing large acoustic datasets [74]. Supervised source separation requires recordings with clean, ground truth-source signals, while unsupervised source separation requires data mixtures [75,76]. Machine Learning applications are growing quickly in the field of ecoacoustics and acoustic community ecology and can, for example, be applied to unseen data as a tool to help bioacousticians identify recurrent sounds and save time when studying their temporo-spatial patterns [73]; however, at the current state of art, ML is not yet completely independent from manual validation. Therefore, manual analysis is relevant and important for providing, for example, labelled data series to train ML semi-supervised algorithms. Manual analysis such as that performed in this study is extremely labor intensive and therefore it must be carried out on a subsample of data. The subsampling chosen for this study was validated in other environments and is applied commonly to fish acoustic community characterisation [50,54]. While not perfect, it represents the best compromise between operator effort and significance of presented results. Future studies are encouraged to identify which manual scrolling subsampling scheme best informs EIAs in the North Sea.

Another bottleneck in the use of PAM for ecological and communities monitoring relates to the type of metrics used to describe status and dynamics of the biological community object of interest. In fish at least, sound types appear as a ‘unit’ capable of providing high resolution information on fish communities status and dynamics. Fish call rates have been proven to be predictors of hard coral cover, fish abundance and fish species richness, while most acoustic indices failed to parse out fine distinctions [77]. A combination of recent ML applications such as those developed by Parcerisas et al. [73], used in conjunction with manual analysis and validation, and potential source identification as attempted in this study, could be a potent combination for successfully applying the eco and bioacoustics paradigm to Environmental Impact Assessments.

Finally, source identification remains a challenge in most environments. Despite decades of research, documentation of underwater sonifery across different taxa remains highly variable [59]. The majority of cetaceans, for example, have been confirmed to produce sounds [see 78 for a comprehensive world list of marine-mammal vocalisations]; in contrast, marine invertebrates and fish and have been relatively less studied when considering the thousands of extant species [59]. In 2021, the Global Inventory of Fish Sounds was launched [60]. Without this database, inferring potential sound-emitting species recorded during this study would have not being possible; therefore, this study strongly supports the importance of shared, open-access repositories of known and unknown sound types, in accordance with Parsons et al. [8] and Looby et al. [60], both in fundamental research and in environmental management.

## Supporting information

S1 Table Soniferous fish in the North Sea.
(XLSX)

S1 Fig PCA graph of variables.
(TIF)

S2 Fig Scree plot.
(TIF)

S1. File FishDetectionData.
(CSV)

S2 File FishSoundMeasurementData.
(CSV)

S3 File PCA_Data.
(CSV)

## References

[1] Farina A. Soundscape ecology: principles, patterns, methods and applications. Media. SSB, editor. 2013.

[2] Krause BL. The great animal orchestra: finding the origins of music in the world’s wild places. Cartier F, editor. New York. 2012.

[3] Gibb R, Browning E, Glover‐Kapfer P, Jones KE. Emerging opportunities and challenges for passive acoustics in ecological assessment and monitoring. Methods Ecol Evol. 2018;10(2):169–85. doi: 10.1111/2041-210x.13101

[4] Mooney TA, Di Iorio L, Lammers M, Lin T-H, Nedelec SL, Parsons M, et al. Listening forward: approaching marine biodiversity assessments using acoustic methods. R Soc Open Sci. 2020;7(8):201287. doi: 10.1098/rsos.201287 32968541 PMC7481698

[5] Lin T, Kawagucci S. Acoustic twilight: A year‐long seafloor monitoring unveils phenological patterns in the abyssal soundscape. Limnology and Oceanography Letters. 2023;2023:1–10.

[6] Ross S, O’Connell D, Deichmann J, Desjonquères C, Gasc A, Phillips J. Passive acoustic monitoring provides a fresh perspective on fundamental ecological questions. Functional Ecology. 2023;37(4):959–75.

[7] Darras KF, Rountree R, Van Wilgenburg S, Dong L, Gasc A, Chen Y, et al. Worldwide soundscape ecology patterns across realms. 2024;(4).

[8] Parsons M, Lin T, Mooney T, Erbe C, Juanes F, Lammers M. Sounding the call for a global library of underwater biological sounds. Frontiers in Ecology and Evolution. 2022;39(10):810156.

[9] Miksis-Olds JL, Bradley DL, Niu XM. Decadal trends in Indian Ocean ambient sound. J Acoust Soc Am. 2013;134(5):3464–75. doi: 10.1121/1.4821537 24180757

[10] Miksis-Olds JL, Madden LE. The power of acoustics in ocean observing systems: A case study in the Bering Sea. J Acoust Soc Am. 2012;132(3):1915.

[11] Leiper A, editor. What does the consultancy industry need from Academia? A soundscape and planning perspective. Acoustic. 2020;2020.

[12] Montgomery JC, Jeffs A, Simpson SD, Meekan M, Tindle C. Sound as an orientation cue for the pelagic larvae of reef fishes and decapod crustaceans. Adv Mar Biol. 2006;51 143–96. doi: 10.1016/S0065-2881(06)51003-X 16905427

[13] Radford C, Jeffs A, Montgomery J. Directional swimming behaviour by five species of crab postlarvae in response to reef sound. Bulletin of Marine Science. 2007;80(2):10.

[14] McKibben JR, Bass AH. Behavioral assessment of acoustic parameters relevant to signal recognition and preference in a vocal fish. J Acoust Soc Am. 1998;104(6):3520–33. doi: 10.1121/1.423938 9857511

[15] Rowe S, Hutchings JA. Sound Production by Atlantic Cod during Spawning. Trans Am Fish Soc. 2006;135(2):529–38. doi: 10.1577/t04-061.1

[16] Gannon DP, Barros NB, Nowacek DP, Read AJ, Waples DM, Wells RS. Prey detection by bottlenose dolphins, *Tursiops truncatus*: an experimental test of the passive listening hypothesis. Animal Behaviour. 2005;69(3):709–20. doi: 10.1016/j.anbehav.2004.06.020

[17] Jordao J, Fonseca P, Amorim M. Chorusing behaviour in the Luistanian Toadfish: should I match my neighbours’ calling rate?. International Journal of Behavioural Biology. 2012;118(9):10.

[18] Simpson SD, Radford AN, Tickle EJ, Meekan MG, Jeffs AG. Adaptive avoidance of reef noise. PLoS One. 2011;6(2):e16625. doi: 10.1371/journal.pone.0016625 21326604 PMC3033890

[19] Amorim MCP. Diversity of sound production in fish. In: Springer, editor. Communication in fishes. 2006. p. 71–104.

[20] Amorim M, Vasconcelos R, Fonseca P. Fish sounds and mate choice. In: Springer, editor. Sound communication in fishes. 2015;1–33.

[21] Parmentier E, Diogo R, Fine ML. Multiple exaptations leading to fish sound production. Fish and Fisheries. 2017;18(5):958–66. doi: 10.1111/faf.12217

[22] Rice AN, Farina SC, Makowski AJ, Kaatz IM, Lobel PS, Bemis WE, et al. Evolutionary patterns in sound production across fishes. Ichthyology & Herpetology. 2022;110(1):1–12. doi: 10.1643/i2020172

[23] Tricas T, Boyle K. Acoustic behaviors in Hawaiian coral reef fish communities. Marine Ecology Progress Series. 2014;511(1):1–16.

[24] Lindseth AV, Lobel PS. Underwater soundscape monitoring and fish bioacoustics: a review. Fishes. 2018;3(3):36. doi: 10.3390/fishes3030036

[25] Desiderà E, Guidetti P, Panzalis P, Navone A, Valentini-Poirrier C, Boissery P, et al. Acoustic fish communities: sound diversity of rocky habitats reflects fish species diversity. Mar Ecol Prog Ser. 2019;608:183–97. doi: 10.3354/meps12812

[26] Bertucci F, Maratrat K, Berthe C, Besson M, Guerra AS, Raick X, et al. Local sonic activity reveals potential partitioning in a coral reef fish community. Oecologia. 2020;193(1):125–34. doi: 10.1007/s00442-020-04647-3 32285197

[27] Carriço R, Silva MA, Menezes GM, Vieira M, Bolgan M, Fonseca PJ, et al. Temporal dynamics in diversity patterns of fish sound production in the Condor seamount (Azores, NE Atlantic). Deep Sea Research Part I: Oceanographic Research Papers. 2020;164 103357. doi: 10.1016/j.dsr.2020.103357

[28] Rountree RA, Juanes F, Bolgan M. Temperate freshwater soundscapes: A cacophony of undescribed biological sounds now threatened by anthropogenic noise. PLoS One. 2020;15(3):e0221842. doi: 10.1371/journal.pone.0221842 32187194 PMC7080229

[29] Sertlek HÖ, Slabbekoorn H, Ten Cate C, Ainslie MA. Source specific sound mapping: Spatial, temporal and spectral distribution of sound in the Dutch North Sea. Environ Pollut. 2019;247:1143–57. doi: 10.1016/j.envpol.2019.01.119 30823343

[30] Basan F, Fischer J-G, Putland R, Brinkkemper J, de Jong CAF, Binnerts B, et al. The underwater soundscape of the North Sea. Mar Pollut Bull. 2024;198:115891. doi: 10.1016/j.marpolbul.2023.115891 38101054

[31] Putland RL, de Jong CAF, Binnerts B, Farcas A, Merchant ND. Multi-site validation of shipping noise maps using field measurements. Mar Pollut Bull. 2022;179:113733. doi: 10.1016/j.marpolbul.2022.113733 35594641

[32] Dol H, Ainslie M, de Jong C, Blacquière G, van Riet M. Natural and anthropogenic sources of sound in the North Sea. The Netherlands; 2009.

[33] von Benda-Beckmann AM, Aarts G, Sertlek HÖ, Lucke K, Verboom WC, Kastelein RA, et al. Assessing the impact of underwater clearance of unexploded ordnance on harbour porpoises (*Phocoena phocoena*) in the southern North Sea. Aquatic Mammals. 2015;41(4):503.

[34] Todd VLG. Mitigation of underwater anthropogenic noise and marine mammals: the “death of a thousand” cuts and/or mundane adjustment?. Mar Pollut Bull. 2016;102(1):1–3. doi: 10.1016/j.marpolbul.2015.11.059 26795130

[35] McKenna MF, Ross D, Wiggins SM, Hildebrand JA. Underwater radiated noise from modern commercial ships. J Acoust Soc Am. 2012;131(1):92–103. doi: 10.1121/1.3664100 22280574

[36] Parsons M, Erbe C, Meekan M, Parsons S. A review and meta-analysis of underwater noise radiated by small (<25 m length) vessels. Journal of Marine Science and Engineering. 2021;827(9):29.

[37] Javier RF, Jaime R, Pedro P, Jesus C, Enrique S. Analysis of the underwater radiated noise generated by hull vibrations of the ships. Sensors (Basel). 2023;23(2):1035. doi: 10.3390/s23021035 36679833 PMC9861438

[38] Todd VLG, Williamson LD, Jiang J, Cox SE, Todd IB, Ruffert M. Proximate underwater soundscape of a North Sea offshore petroleum exploration jack-up drilling rig in the Dogger Bank. J Acoust Soc Am. 2020;148(6):3971. doi: 10.1121/10.0002958 33379917

[39] Todd VLG, Williamson LD, Cox SE, Todd IB, Macreadie PI. Characterizing the first wave of fish and invertebrate colonization on a new offshore petroleum platform. ICES Journal of Marine Science. 2019;77(3):1127–36. doi: 10.1093/icesjms/fsz077

[40] Todd VLG, Susini I, Williamson LD, Todd IB, McLean DL, Macreadie PI. Characterizing the second wave of fish and invertebrate colonization of an offshore petroleum platform. ICES Journal of Marine Science. 2021;78(3):1131–45. doi: 10.1093/icesjms/fsaa245

[41] Todd V, Williamson L, Couto A, Todd I, Clapham P. Effect of a new offshore gas platform on harbor porpoise in the Dogger Bank. Marine Mammal Science. 2022;38(4):1609–22.

[42] Todd VLG, Warley JC, Williamson LD, Todd IB. Acoustic activity of harbour porpoise around an offshore oil and gas platform. The Effects of Noise on Aquatic Life: Springer; 2024. p. 17.

[43] Hawkins AD, Picciulin M. The importance of underwater sounds to gadoid fishes. Journal of the Acoustical Society of America. 2019;146(5). 10.1121/1.5134683 31795661

[44] Aguzzi J, López-Romero D, Marini S, Costa C, Berry A, Chumbinho R, et al. Multiparametric monitoring of fish activity rhythms in an Atlantic coastal cabled observatory. Journal of Marine Systems. 2020;212:103424. doi: 10.1016/j.jmarsys.2020.103424

[45] Fish MP, Mowbray WH. Sounds of Western North Atlantic fishes. A reference file of biological underwater sounds. Rhode Island Universirty of Kingston Narragansett Marine Laboratory; 1970.

[46] Fugro. Geophysical site and route survey block A18 A18 site survey (geotechnical) a18 platform site Netherlands continental shelf, North Sea. Survey Period: July 2013. Report Number: GH106-R4. SSC-UN-13017. FUGRO SURVEY B.V. Leidschendam, The Netherlands: Prepared for Chevron E&P Netherlands B.V.; 2013.

[47] Fugro. Annual Pipeline Inspection Survey 2015 Dutch Continental Shelf, North Sea Block A A18 to A12-CPP 12 inch gas pipeline June 2015 Fugro Project No.: GH146-R2/ U11 Client Reference: SSC-UN-13025. Leidschendam, The Netherlands: Prepared for Petrogas E&P Netherlands B.V.; 2015.

[48] Cato DH. Marine biological choruses observed in tropical waters near Australia. The Journal of the Acoustical Society of America. 1978;64(3):736–43. doi: 10.1121/1.382038

[49] White P. Long-term average spectrum (LTAS) analysis of sex- and gender-related differences in children’s voices. Logoped Phoniatr Vocol. 2001;26(3):97–101. doi: 10.1080/14015430152728007 11824501

[50] Bolgan M, Di Iorio L, Dailianis T, Catalan IA, Lejeune P, Picciulin M, et al. Fish acoustic community structure in Neptune seagrass meadows across the Mediterranean basin. Aquatic Conservation. 2022;32(2):329–47. doi: 10.1002/aqc.3764

[51] Merchant ND, Fristrup KM, Johnson MP, Tyack PL, Witt MJ, Blondel P, et al. Measuring acoustic habitats. Methods Ecol Evol. 2015;6(3):257–65. doi: 10.1111/2041-210X.12330 25954500 PMC4413749

[52] Bolgan M, Gervaise C, Di Iorio L, Lossent J, Lejeune P, Raick X, et al. Fish biophony in a Mediterranean submarine canyon. J Acoust Soc Am. 2020;147(4):2466. doi: 10.1121/10.0001101 32359295

[53] Di Iorio L, Audax M, Deter J, Holon F, Lossent J, Gervaise C, et al. Biogeography of acoustic biodiversity of NW Mediterranean coralligenous reefs. Sci Rep. 2021;11(1):16991. doi: 10.1038/s41598-021-96378-5 PMC8379277 34417502

[54] Puebla-Aparicio M, Ascencio-Elizondo C, Vieira M, Amorim M, Duarte R, Fonseca P. Characterization of the fish acoustic communities in a Mozambican tropical coral reef. Mar Ecol Prog Ser. 2024;727:143–58. doi: 10.3354/meps14450

[55] Raick X, Di Iorio L, Lecchini D, Gervaise C, Hedouin L, Under the Pole Consortium. Fish sounds of photic and mesophotic coral reefs: variation with depth and type of island. Coral Reefs. 2023;42(2):13.

[56] La Manna G, Merella M, Vargiu R, Morello G, Sara G, Ceccherelli G. Spatio-temporal patterns of fish acoustic communities in Western Mediterranean coralligenous reefs: optimizing monitoring through recording duration. Frontiers in Marine Science. 2024;14.

[57] Fine ML, Parmentier E. Mechanisms of fish sound production. Sound communication in fishes. Springer-Verlag; 2015. p. 77–126

[58] Parmentier E, Fine ML. Fish sound production: insights. Vertebrate sound production and acoustic communication. Springer International Publishing; 2016, p. 19–49.

[59] Looby A, Erbe C, Bravo S, Cox K, Davies HL, Di Iorio L, et al. Global inventory of species categorized by known underwater sonifery. Sci Data. 2023;10(1):892. doi: 10.1038/s41597-023-02745-4 38110417 PMC10728183

[60] Looby A, Vela S, Cox K, Riera A, Bravo S, Davies H, et al. FishSounds Version 1.0: A website for the compilation of fish sound production information and recordings. Ecol Inform. 2023;74:101953.

[61] Halpern BS, Walbridge S, Selkoe KA, Kappel CV, Micheli F, D’Agrosa C, et al. A global map of human impact on marine ecosystems. Science. 2008;319(5865):948–52. doi: 10.1126/science.1149345 18276889

[62] Rako-Gospić N, Picciulin M. Chapter 20 - Underwater noise: sources and effects on marine life. In: Sheppard C, editor. World Seas: an Environmental Evaluation (Second Edition). Academic Press; 2019. p. 367–89.

[63] Amorim MCP, Vieira M, Meireles G, Novais SC, Lemos MFL, Modesto T, et al. Boat noise impacts Lusitanian toadfish breeding males and reproductive outcome. Sci Total Environ. 2022;830:154735. doi: 10.1016/j.scitotenv.2022.154735 35337882

[64] Faria A, Fonseca PJ, Vieira M, Alves LMF, Lemos MFL, Novais SC, et al. Boat noise impacts early life stages in the Lusitanian toadfish: A field experiment. Sci Total Environ. 2022;811:151367. doi: 10.1016/j.scitotenv.2021.151367 34740663

[65] Stenton CA, Bolger EL, Michenot M, Dodd JA, Wale MA, Briers RA, et al. Effects of pile driving sound playbacks and cadmium co-exposure on the early life stage development of the Norway lobster, *Nephrops norvegicus*. Mar Pollut Bull. 2022;179:113667. doi: 10.1016/j.marpolbul.2022.113667 35533617

[66] Staaterman E, Paris C, DeFerrari H, Mann D, Rice A, D’Alessandro E. Celestial patterns in marine soundscapes. Mar Ecol Prog Ser. 2014;508:17–32. doi: 10.3354/meps10911

[67] Ceraulo M, Papale E, Caruso F, Filiciotto F, Grammauta R, Parisi I, et al. Acoustic comparison of a patchy Mediterranean shallow water seascape: Posidonia oceanica meadow and sandy bottom habitats. Ecological Indicators. 2018;85:1030–43. doi: 10.1016/j.ecolind.2017.08.066

[68] Fox CJ, Taylor M, Dickey-Collas M, Fossum P, Kraus G, Rohlf N, et al. Mapping the spawning grounds of North Sea cod (*Gadus morhua*) by direct and indirect means. Proc Biol Sci. 2008;275(1642):1543–8. doi: 10.1098/rspb.2008.0201 18397869 PMC2602663

[69] Caiger P, Dean M, DeAngelis A, Hatch L, Rice A, Stanley J, et al. A decade of monitoring Atlantic cod Gadus morhua spawning aggregations in Massachusetts Bay using passive acoustics. Mar Ecol Prog Ser. 2020;635:89–103. doi: 10.3354/meps13219

[70] Amorim MCP, Stratoudakis Y, Hawkins AD. Sound production during competitive feeding in the grey gurnard. J Fish Biol. 2004;65(1):182–94. doi: 10.1111/j.0022-1112.2004.00443.x

[71] Picciulin M, Bolgan M, Codarin A, Fiorin R, Zucchetta M, Malavasi S. Passive acoustic monitoring of *Sciaena umbra* on rocky habitats in the Venetian littoral zone. Fish Research. 2013;145:76–81.

[72] Di Iorio L, Raick X, Parmentier E, Boissery P, Valentini-Poirier CA, Gervaise C. ‘*Posidonia* meadows calling’: a uniqiotous fish sound with monitoring potential. Remote Sensing in Ecology and Conservation. 2017;1–16.

[73] Parcerisas C, Schall E, te Velde K, Botteldooren D, Devos P, Debusschere E. Machine learning for efficient segregation and labeling of potential biological sounds in long-term underwater recordings. Front Remote Sens. 2024;5:1390687. doi: 10.3389/frsen.2024.1390687

[74] Nieto-Mora DA, Rodríguez-Buritica S, Rodríguez-Marín P, Martínez-Vargaz JD, Isaza-Narváez C. Systematic review of machine learning methods applied to ecoacoustics and soundscape monitoring. Heliyon. 2023;9(10):e20275. doi: 10.1016/j.heliyon.2023.e20275 37790981 PMC10542774

[75] Neri J, Badeau R, Depalle P, auto-encoders Ubsswv. Unsupervised blind source separation with variational auto-encoders. In: IEEE., editor. 29th European Signal Processing Conference (EUSIPCO). 2021, 311–5.

[76] Calonge A, Parcerisas C, Schall E, Debusschere E. Revised clusters of annotated unknown sounds in the Belgian part of the North Sea. Frontiers in Remote Sensing. 2024;5:1384562.

[77] Jarriel SD, Formel N, Ferguson SR, Jensen FH, Apprill A, Mooney TA. Unidentified fish sounds as indicators of coral reef health and comparison to other acoustic methods. Front Remote Sens. 2024;5:1338585. doi: 10.3389/frsen.2024.1338586

[78] Todd V, Todd I, Gardiner J, Morrin E. Marine mammal observer and passive acoustic monitoring handbook. Exeter, UK: Pelagic Publishing Ltd; 2015.

