# Supplementary figures and images for: Soundscape and fish passive acoustic monitoring around a North Sea gas-production platform in the Dogger Bank
Source: PLoS One. 2025 Apr 2;20(4):e0319536. doi: 10.1371/journal.pone.0319536 (PMC11964218; doi:10.1371/journal.pone.0319536)

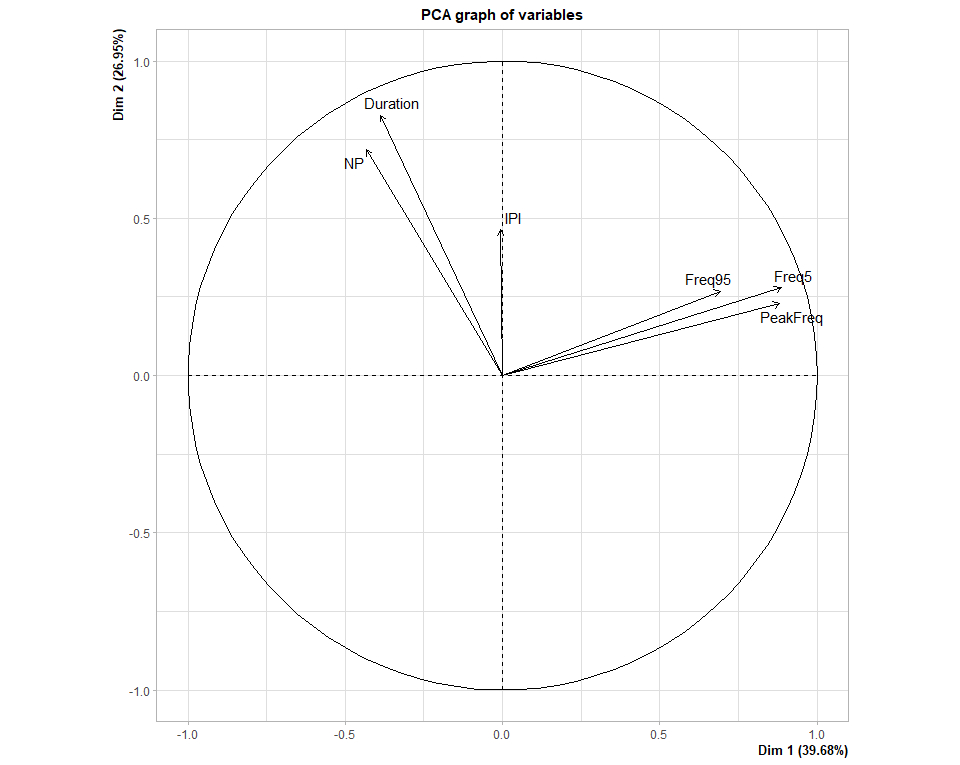

Supplement: S1 Fig — (TIF) [file pone.0319536.s002.tif]

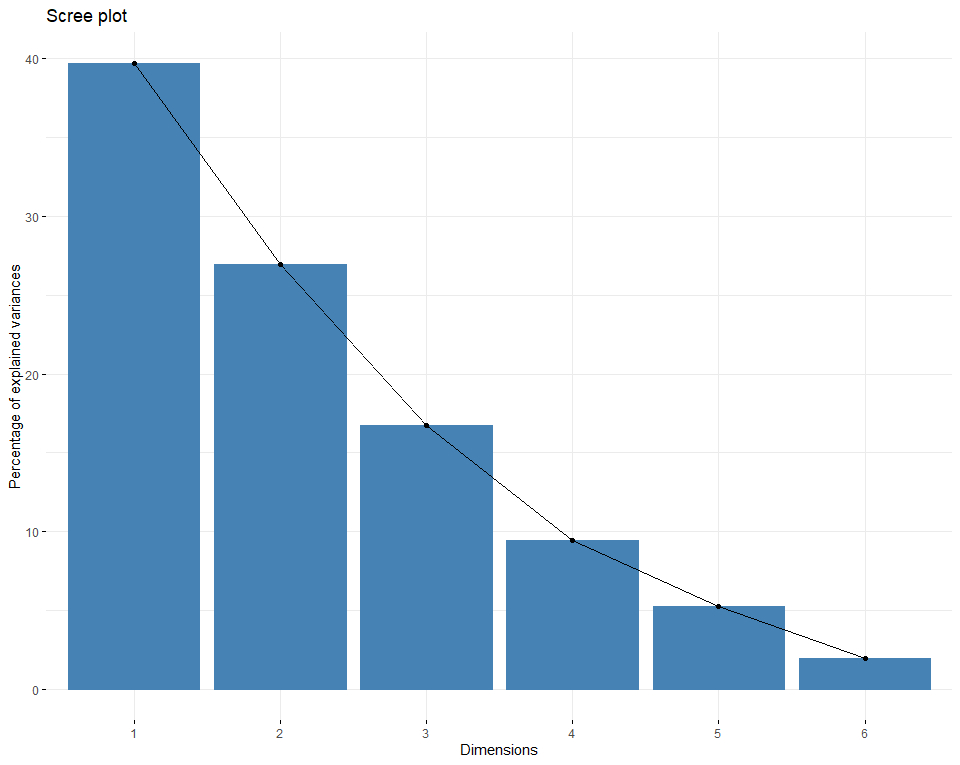

Supplement: S2 Fig — (TIF) [file pone.0319536.s003.tif]
